# Supplementary material for: Optimal blade pitch control for enhanced vertical-axis wind turbine performance
Source: Nat Commun. 2024 Mar 30;15:2770. doi: 10.1038/s41467-024-46988-0 (PMC10980684; doi:10.1038/s41467-024-46988-0)
Supplement: Supplementary file 1 — Supplementary information [file 41467_2024_46988_MOESM1_ESM.pdf]

# Supplementary information: Optimal blade pitch control for enhanced vertical-axis wind turbine performance

Sébastien Le Fouest[1] and Karen Mulleners[1,*]

[1]*Institute of Mechanical Engineering, École Polytechnique Fédérale de Lausanne (EPFL), Lausanne, CH-1015, Switzerland*
[*]*Corresponding author: karen.mulleners@epfl.ch*

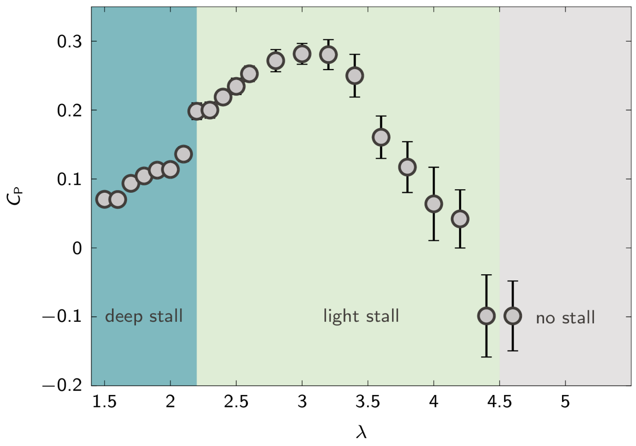


**Supplementary Figure S1. Power coefficient as a function of tip-speed ratio for the single-blade wind turbine model.** The expected stall regimes given the wind turbine's geometry are shown in the background and were obtained from the theoretical parametric dynamic stall regime map given in Le Fouest et al. (2022).

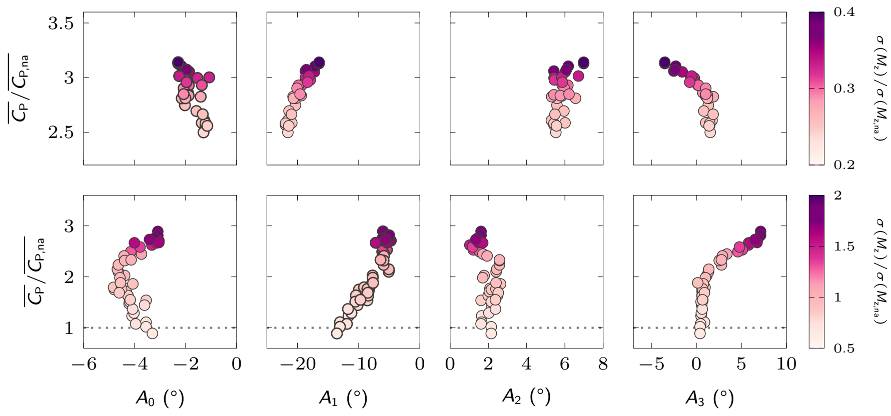


**Supplementary Figure S2. Optimal pitching kinematics: the role of pitching amplitude for both wind conditions.** Variations of the mean offset angle ($A_0$) and amplitudes of the three harmonics ($A_1$ - $A_3$) of the Pareto-optimal pitching kinematics for $\lambda = 1.5$ (top row) and $\lambda = 3.2$ (bottom row).

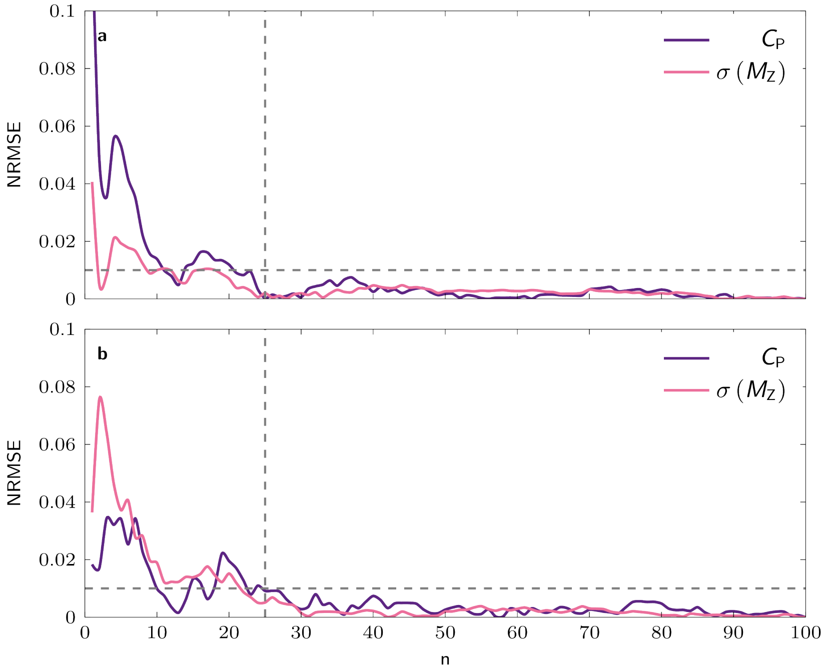


**Supplementary Figure S3. Convergence analysis of the mean value for both optimisation objectives.** Evolution of the normalised root mean square error (NRMSE) for both objective functions (power coefficient $C_\mathrm{P}$ and pitching moment standard deviation $\sigma(M_\mathrm{Z})$) with the number of turbine rotations $n$ for tip-speed ratios **a** $\lambda = 1.5$ and **b** $\lambda = 3.2$. The RMSE is normalised using the mean value obtained for the objective functions over 100 turbine rotations. After 25 rotations both the power coefficient $C_\mathrm{P}$ and the pitching moment standard deviation $\sigma(M_\mathrm{Z})$ have a NRMSE below 1 %.

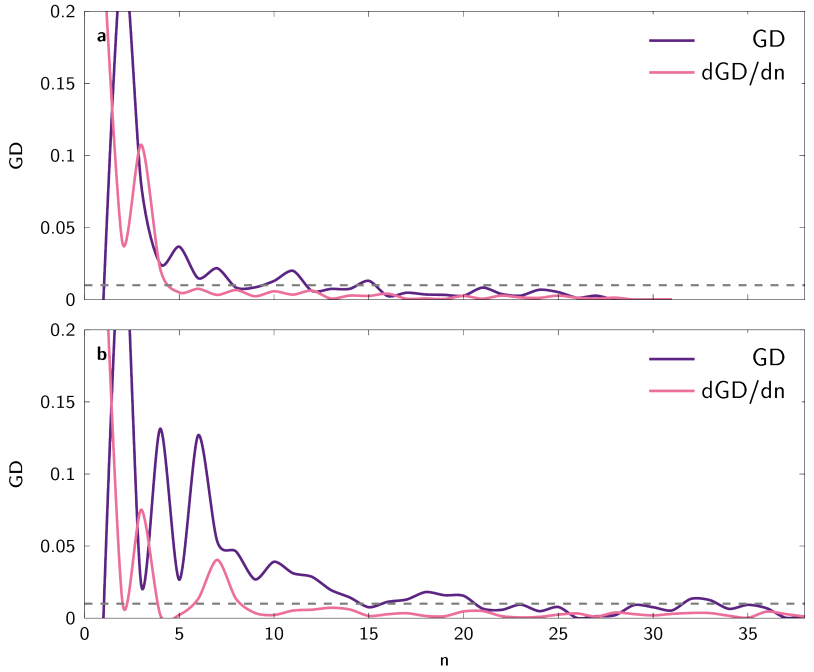


**Supplementary Figure S4. Convergence analysis of the optimisation procedure for both wind conditions.** Evolution of the generational distance (GD) and its gradient as a function of the number of generations (n) tested by the genetic algorithm for optimisation at **a** $\lambda = 1.5$ and **b** $\lambda = 3.2$. The generational distance is a measure for the convergence of the Pareto front. The dashed line is at a generational distance of 1 %.

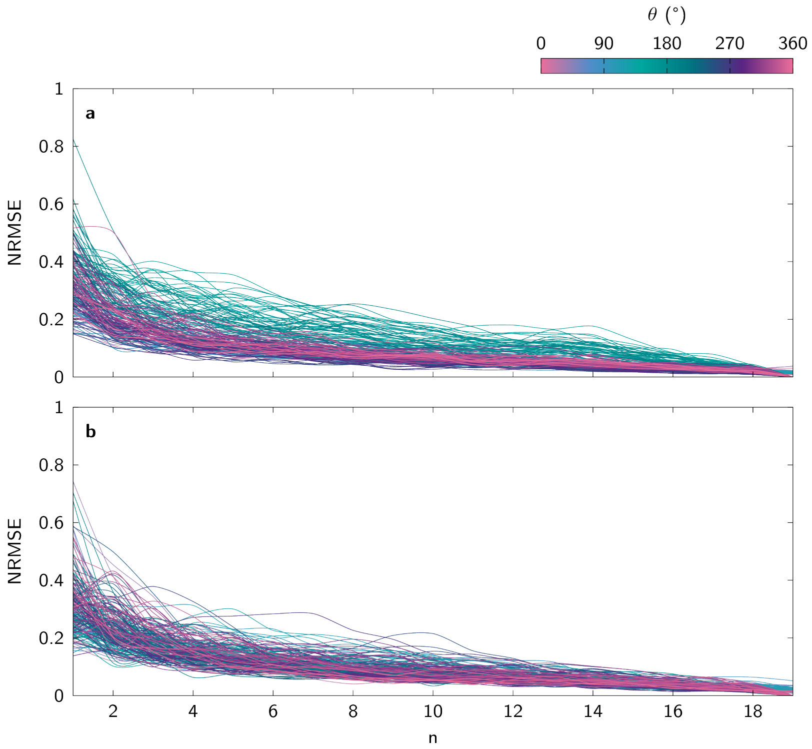


**Supplementary Figure S5. Convergence analysis of the phase-averaged particle image velocimetry for all phases.** Root mean square error between the phase averaged velocity magnitude field calculated over all 19 cycles and the phase averaged velocity magnitude field over $n$ cycles as a function of the number of cycles for 200 individual phase bins. The root mean square error is normalised by the characteristic blade velocity to obtain a normalised root mean square error (NRMSE). Results are shown for **a** $\lambda = 1.5$ and **b** $\lambda = 3.2$, respectively. The phase averages converge earlier for the higher tip speed ratio case and for $0° \leq \theta < 90°$ and $270° < \theta \leq 360°$, when the flow is attached during the lower tip speed ratio case. For the phases where the flow is separated, the NRMSE is larger than for the phases that correspond to attached flow. Overall, we consider to have reached acceptable convergence after 10 to 15 cycles.

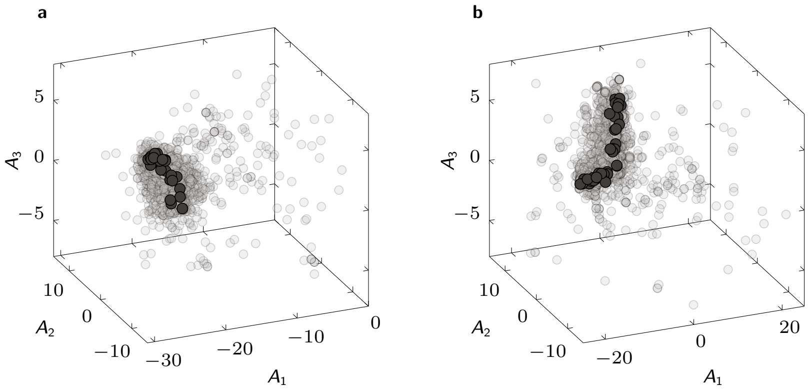


**Supplementary Figure S6. Distribution of the Fourrier coefficients within the optimisation search space bounds for both wind conditions.** Each tested individual is represented by a light symbol and Pareto-optimal individuals are represented by a dark symbol for optimisation at **a** $\lambda = 1.5$ and **b** $\lambda = 3.2$. The search bound are specified in **Table 1** of the main document. Pareto optimal individuals are far from the domain boundaries, indicating that the search domain was not over-constrained.

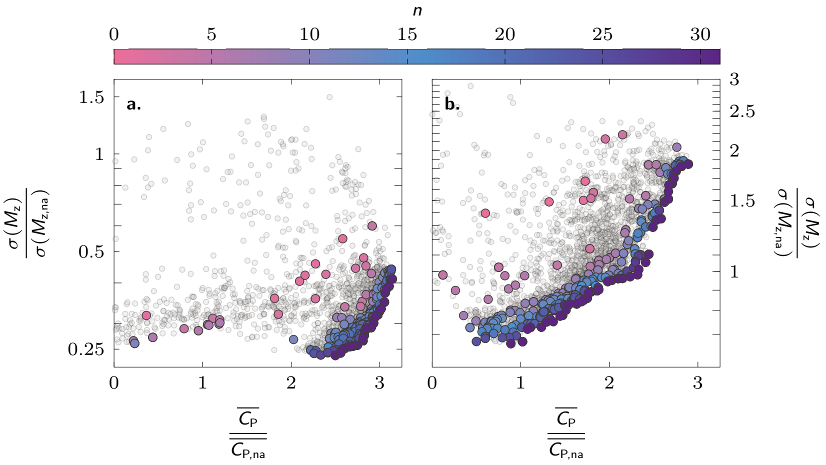


**Supplementary Figure S7. The evolution of the Pareto-optimal individuals colour-coded by generation (n) for the optimisations at a $\lambda = 1.5$ and b $\lambda = 3.2$.**
